# Rehabilitation Supported by Technology: Protocol for an International Cocreation and User Experience Study

**DOI:** 10.2196/34537

**Published:** 2022-03-10

**Authors:** Sylvie Bernaerts, Nele A J De Witte, Vicky Van der Auwera, Bert Bonroy, Luiza Muraru, Panagiotis Bamidis, Christos Frantzidis, Chrysoula Kourtidou-Papadeli, Nancy Azevedo, Jokin Garatea, Idoia Muñoz, Rosa Almeida, Raquel Losada, Joyce Fung, Eva Kehayia, Anouk Lamontagne, Elaine de Guise, Cyril Duclos, Johanne Higgins, Sylvie Nadeau, Lucie Beaudry, Evdokimos Konstantinidis

**Affiliations:** 1 LiCalab Thomas More University of Applied Sciences Geel Belgium; 2 Expertise Unit Psychology, Technology & Society Thomas More University of Applied Sciences Antwerpen Belgium; 3 Mobilab & Care Thomas More University of Applied Sciences Geel Belgium; 4 Medical Physics and Digital Innovation Laboratory School of Medicine Aristotle University of Thessaloniki Thessaloniki Greece; 5 Greek Aerospace Medical Association and Space Research Thessaloniki Greece; 6 Centre de recherche interdisciplinaire en réadaptation du Montréal métropolitain Institut Universitaire sur la réadaptation en déficience physique de Montréal Montreal, QC Canada; 7 GAIA Asociación de Industrias de Conocimiento y Tecnologías Aplicadas San Sebastian Spain; 8 Fundación INTRAS Research, Development and Innovation Department Valladolid Spain; 9 School of Physical and Occupational Therapy McGill University Montreal, QC Canada; 10 Université de Montreal Montreal, QC Canada; 11 Dance Department Université du Québec à Montréal Montreal, QC Canada; 12 European Network of Living Labs Brussels Belgium

**Keywords:** cocreation, harmonization, living lab, rehabilitation, small-scale real-life testing, technology

## Abstract

**Background:**

Living labs in the health and well-being domain have become increasingly common over the past decade but vary in available infrastructure, implemented study designs, and outcome measures. The Horizon 2020 Project *Virtual Health and Wellbeing Living Lab Infrastructure* aims to harmonize living lab procedures and open living lab infrastructures to facilitate and promote research activities in the health and well-being domain in Europe and beyond. This protocol will describe the design of a joint research activity, focusing on the use of innovative technology for both rehabilitation interventions and data collection in a rehabilitation context.

**Objective:**

With this joint research activity, this study primarily aims to gain insight into each living lab’s infrastructure and procedures to harmonize health and well-being living lab procedures and infrastructures in Europe and beyond, particularly in the context of rehabilitation. Secondarily, this study aims to investigate the potential of innovative technologies for rehabilitation through living lab methodologies.

**Methods:**

This study has a mixed methods design comprising multiple phases. There are two main phases of data collection: cocreation (phase 1) and small-scale pilot studies (phase 2), which are preceded by a preliminary harmonization of procedures among the different international living labs. An intermediate phase further allows the implementation of minor adjustments to the intervention or protocol depending on the input that was obtained in the cocreation phase. A total of 6 small-scale pilot studies using innovative technologies for intervention or data collection will be performed across 4 countries. The target study sample comprises patients with stroke and older adults with mild cognitive impairment. The third and final phases involve Delphi procedures to reach a consensus on harmonized procedures and protocols.

**Results:**

Phase 1 data collection will begin in March 2022, and phase 2 data collection will begin in June 2022. Results will include the output of the cocreation sessions, small-scale pilot studies, and advice on harmonizing procedures and protocols for health and well-being living labs focusing on rehabilitation.

**Conclusions:**

The knowledge gained by the execution of this research will lead to harmonized procedures and protocols in a rehabilitation context for health and well-being living labs in Europe and beyond. In addition to the harmonized procedures and protocols in rehabilitation, we will also be able to provide new insights for improving the implementation of innovative technologies in rehabilitation.

**International Registered Report Identifier (IRRID):**

PRR1-10.2196/34537

## Introduction

### Background

#### Virtual Health and Wellbeing Living Lab Infrastructure

Living labs in the health and well-being domains have become increasingly common over the past decade but vary in available infrastructure, implemented study designs, and outcome measures. Therefore, increased transnational collaboration and harmonization can further improve research quality. The Horizon 2020 Project *Virtual Health and Wellbeing Living Lab Infrastructure* (VITALISE), funded by the European Union (under grant agreement 101007990; April 2021 to March 2024), unites 19 partners (AIT, AUTH, AV, CERTH, ENoLL, GAIA, INTRAS, LAUREA, LiCalab, LLSA, McGILL, SLIMMER, SIT, TREBAG, UdeM, UPM, VICOM, VILABS, and WITA) across 11 countries (Table 1). VITALISE aims to harmonize living lab procedures and open living lab infrastructures as a means to facilitate and promote research activities in the health and well-being domain in Europe and beyond. To do so, the VITALISE consortium will conduct joint research activities (JRAs) in the fields included in the care pathway of patients, namely, rehabilitation, transitional care, and everyday living environments for the older adults. These JRAs combine and capitalize on research experience and expertise from the different living labs in the consortium and create innovation test beds for the harmonized procedures and infrastructures in the context of health and well-being research. This protocol will describe the design of the rehabilitation JRA, focusing on the use of innovative technology for both rehabilitation interventions and data collection in a rehabilitation context. More information about the project can be found on the project website [1].

**Table 1:** List of partners in the VITALISE^a^ project.

Abbreviation	Full (legal) name	Location
AIT	Austrian Institute of Technology GmbH	Austria
AUTH^b^	Aristotelio Panepistimio Thessalonikis	Greece
AV	Anthology Venture Ad	Bulgaria
CERTH	Center for Research and Technology Hellas (Ethniko Kentro Erevnas Kai Technologikis Anaptyxis)	Greece
CRIR^c^	Centre for Interdisciplinary Research in Rehabilitation of Greater Montreal	Canada
ENoLL	European Network of Living Labs	Belgium
GAIA^b^	Association of Electronic and Information Technologies in the Basque Country	Spain
INTRAS^b^	Fundación INTRAS	Spain
LAUREA	Laurea-ammatikorkeakoulu Oy	Finland
LiCalab^b^	Living & Care Lab (Thomas More Kempen vzw)	Belgium
LLSA	Le Forum des Living Labs en Santé et Autonomie	France
McGILL^b^	McGill University	Canada
SLIMMER	Coöperatie Slimmer Leven 2020 U.A.	The Netherlands
SIT	Social IT Software & Consulting srl	Italy
TREBAG^b^	TREBAG Intellectual Property and Project Manager Ltd	Hungary
UdeM^b^	Université de Montreal	Canada
UQAM^c^	Université de Québec à Montréal	Canada
UPM	Universidad Politécnica de Madrid	Spain
VICOM	Fundación Centro De Tecnologías De Interacción Visual y Comunicaciones VICOMTECH	Spain
VILABS	VILABS OE	Greece
WITA	WITA S.r.l.	Italy

^a^VITALISE: Virtual Health and Wellbeing Living Lab Infrastructure.

^b^The living labs involved in the joint research activity.

^c^The living labs and organizations that participate as VITALISE consortium external partners.

#### Living Lab Methodology

Living labs are defined as user-centered, open innovation ecosystems based on a systematic user cocreation approach integrating research and innovation processes in real-life communities and settings. In practice, living labs are research infrastructures that place the citizen at the center of innovation and have thus shown the ability to better exploit the opportunities offered by new information and communication technology concepts and solutions to the specific needs and aspirations of local contexts, cultures, and creativity potentials [2]. In the context of rehabilitation, these infrastructures can range from research labs in universities (of applied sciences), over rehabilitation centers or rehabilitation departments of (health) care facilities, to smart living homes and individual’s home environments. Living labs can thus be defined as open innovation systems that aim to enable innovation through research activities in realistic circumstances with multiple stakeholders from the quadruple helix (university, industry, government, and public actors) [3,4]. They not only provide access to research infrastructures and end user populations to help tailor innovations to the needs of the local context but also facilitate international upscaling of innovations through cross-border research and collaboration within international networks such as the ENoLL. The living lab methodology is grounded in an iterative, agile, and multimethod research approach including activities for exploration, cocreation, and testing and evaluation of innovations [5,6]. Cocreation activities are central to living lab research. Cocreation or co-design sessions are aimed at creating ideas and concepts together in an interactive manner by, for example, using prototypes, wireframes, or other verbal and visual generative tools [7]. Information from cocreation activities can be implemented to improve innovations or their implementation processes. A subsequent step in the iterative living lab approach can consist of lab-based or real-life testing of innovations with end users. This evaluation activity provides further information about the usability, acceptability, and impact of interventions.

#### Technological Innovations in a Rehabilitation Context

For this study (JRA), we will focus on the use of technology for rehabilitation in patients with stroke and people living with mild cognitive impairment (MCI; in accordance with the expertise and available infrastructures of the involved living labs). Stroke is a leading health problem, and its burden is increasing worldwide [8]. Similarly, the prevalence of dementia, a neurodegenerative condition with a high physical, psychological, social, and economic impact for both patients and their carers, is rising sharply [9]. MCI is an intermediate state between normal cognition and dementia with preserved functional abilities [10]. Accumulating evidence supports the use of technology during the rehabilitation process both on a cognitive level and on a physical level. Concerning cognitive rehabilitation, García-Casal et al [11] have shown that computer-based cognitive interventions for people living with dementia lead to (moderate) beneficial effects on cognition, depression, and anxiety. For example, Gradior cognitive rehabilitation is a computer-based program for neuropsychological assessment and cognitive stimulation in healthy individuals as well as for neuropsychological rehabilitation in people with one or more cognitive disorders [12]. Concerning physical rehabilitation, a wide variety of technologies have been proposed, for example, virtual reality (VR), technologies for remote rehabilitation (telerehabilitation), technologies for augmenting gait training, and a short-arm human centrifuge (SAHC) for gravity therapy. A growing body of evidence supports the use of VR for rehabilitation of different pathologies. The benefits of using VR during the rehabilitation process of patients with stroke, patients with cerebral palsy, patients with spinal cord injuries, and other pathologies include improvements in balance and gait [13] and motor functions, greater community participation, and improved psychological and cognitive function [14]. With advances in information and communication technologies, telerehabilitation has gained increased research interest, as it allows rehabilitation services to be provided to patients remotely in their homes or elsewhere [15]. Research has shown that the effectiveness of telerehabilitation for patients with stroke is similar to that of traditional face-to-face rehabilitation but that different stakeholders have different opinions on the subject [16]. Patients are satisfied with telerehabilitation provided that it is appropriate and some social interaction occurs, whereas clinicians prefer face-to-face interactions and will only use telerehabilitation when face-to-face interactions are not feasible [16]. Another example of a promising technology for physical rehabilitation is the use of a split-belt treadmill for balance and gait training in patients with stroke [17,18]. Finally, gravity therapy by means of a SAHC, an integrated multisystem countermeasure to provide artificial gravity training for rehabilitation purposes, has been proposed to have beneficial effects for individuals with neuromuscular disorders, balance disorders, stroke, and sports injuries. By simulating natural gravity, it targets physiological deconditioning imposed by inactivity or a lack of gravitational force. It functions by exerting a centrifugal force on a body accelerated centripetally in a rotating device [15]. However, more research is needed to be able to provide a personalized therapy [19,20].

In addition, technology is used not only during interventions but also for diagnostics, evaluation of intervention effects, and remote monitoring. For example, in the context of the evaluation of physical activity (physical rehabilitation) and stress (mental rehabilitation), wearables are becoming increasingly common. Wearables are a specific type of mobile health application comprising sensors and devices intended to be worn on the body while collecting longitudinal and continuous data on cardiac cycles, electrodermal activity, skin temperature, acceleration, and so on, on a reliable and noninvasive manner outside of lab settings [21]. Also, a variety of wearable technologies for stroke rehabilitation have been studied to improve the diagnosis and treatment of upper-limb impairment (for a review, see the study by Maceira-Elvira et al [22]).

Despite proven effectiveness in research, however, the implementation of these technologies in practice is slow. For example, in the case of VR, barriers to successful adoption in rehabilitation practice involve three categories: technology development (eg, the degree of match or mismatch between the system and the client’s goals or needs), competency development for end users (eg, perceived ease of use and utility), and facilitated clinical implementation (eg, access to technology and support for setup) [23]. Thus, there is a clear need not only to assess the effectiveness of these technology-based interventions but also to involve stakeholders from the design process to the evaluation of these innovations and to include assessments of feasibility, usability, user experience, and user acceptance. Therefore, living lab research methodologies are needed to implement these innovations and technologies in practice more successfully.

### Study Aims and Objectives

VITALISE aims to harmonize living lab procedures and open living lab infrastructures as a means to facilitate and promote research activities in the health and well-being domain in Europe and beyond. With this JRA, we primarily aim to gain insight into each living lab’s infrastructure and procedures to harmonize health and well-being living lab procedures and infrastructures, particularly in the context of rehabilitation. Secondarily, we aim to investigate the potential of innovative technologies for rehabilitation through living lab methodologies. To do so, multiple international living labs will organize cocreation sessions with patients and care professionals to capture stakeholder perspectives on rehabilitation technology (phase 1) and organize separate small-scale pilot studies to test 6 innovative intervention or data collection technologies in practice using preliminary harmonized procedures (phase 2). The living lab research activities have the following objectives: (1) to assess how different stakeholders across multiple countries view the role of technology in rehabilitation; (2) to collect suggestions regarding specific rehabilitation innovations (phase 1); (3) to perform small-scale pilot studies providing insights into end user experiences and usability of 6 selected cognitive and motor rehabilitation interventions; and (4) to explore the effects of these pilot interventions through self-report, physiological, and motor data of small samples (phase 2).

## Methods

### Design

**Figure 1:**
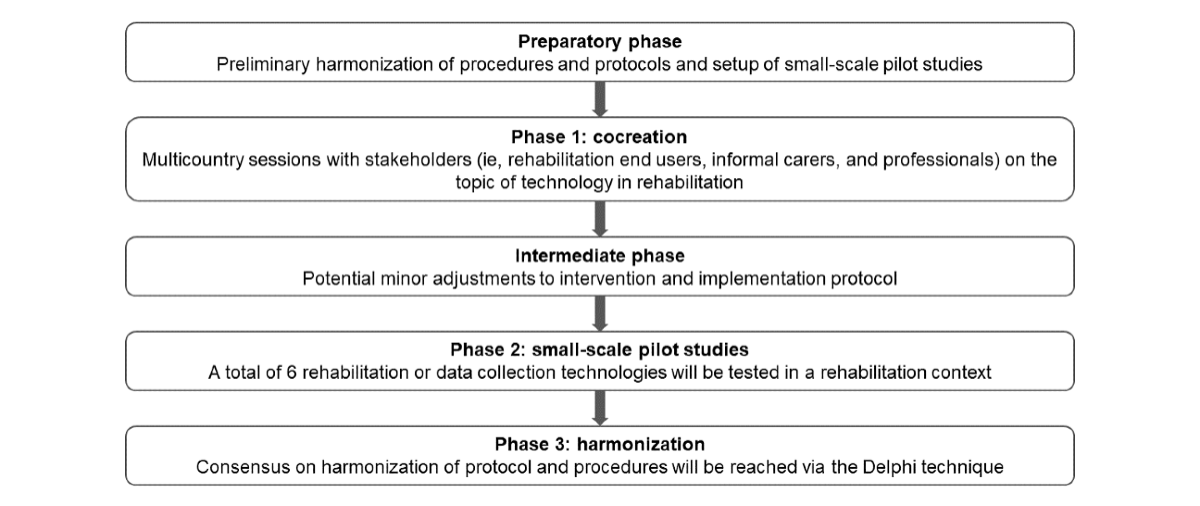
Overview of the protocol design.

This study has a mixed methods design comprising multiple phases (Figure 1) [24]. There are two main phases: phase 1 (cocreation) and phase 2 (small-scale pilot studies), which are preceded by a preliminary harmonization of procedures and protocols among the different international living labs. During this preparatory phase, common interests concerning outcomes, outcome measures, and available infrastructures are discussed among the involved living labs to reach a preliminary harmonization and set up the small-scale pilot studies. In addition, ethical committee applications will be performed by the respective living labs. An intermediate phase (between phases 1 and 2) further allows the implementation of minor adjustments to the intervention or protocol depending on the input that was obtained in cocreation and discussions among living labs. This is in line with an iterative and agile design cycle during which innovations can have different maturity levels and undergo improvements during the research activities. However, adjustments should remain within the limits of the protocol approved by the ethical committee or be submitted as an amendment to this protocol. Note that the intermediate phase might differ among study partners depending on the results of the separate cocreation sessions. The preparatory phase is currently ongoing, and preliminary protocols to implement harmonized living lab procedures and infrastructures are being created.

### Site Selection, Sample, and Recruitment

Study phases 1 and 2 will be conducted by each living lab; therefore, recruitment will be carried out at each living lab’s partner rehabilitation centers, rehabilitation departments of hospitals, or transitional living facilities in the context of rehabilitation across Europe and Canada. Participants will include patients with stroke or individuals with MCI (depending on the technology of interest and the planned small-scale pilot study).

In phase 1 (cocreation), approximately 4 to 12 participants will be included per session in accordance with guidelines for group sessions [25,26]. In phase 2 (small-scale pilot studies), each pilot aims to include approximately 15 participants, as previous research has suggested that this sample size is sufficient to detect most usability problems (up to 90%) [27,28]. Note, however, that the exact numbers can differ among the test sites depending on partner-specific objectives and methods.

### Data Collection

#### Cocreation (Phase 1)

In cocreation, we will bring together rehabilitation end users, informal caregivers, and professionals in sessions to explore their views on technology in rehabilitation in general as well as on the specific technologies used in the small-scale pilot studies. End users are older adults with physical or (mild) cognitive disabilities. Informal caregivers are individuals who provide physical and emotional help to the end users, often close family members or friends, but who are not paid or trained by statutory bodies to do so. Professionals are individuals who work in a rehabilitation setting and can, for example, be clinicians (eg, physicians, physiotherapists, occupational therapists, psychologists, and speech therapists), directors, innovation managers, or other key staff members. Sessions will be organized at six test sites in four countries (Belgium, Canada, Greece, and Spain). Recruitment will be conducted by each regional living lab. The VITALISE project website [29] provides links to living lab hosting organizations home pages where more detailed information about each living lab and their infrastructure is available. Each session will include approximately 8 end users or professionals in accordance with the guidelines for group sessions [25,26]. The cocreation phase will consist of two parts: (1) a general part addressing views on technology in rehabilitation in general and (2) a specific part addressing the technology that will be studied in the small-scale pilot study. Both parts may be addressed in 1 to 4 sessions, depending on the availability (time) of the participants. The cocreation workshop scenario will be provided by LiCalab to direct the cocreation sessions across the different test sites, but the actual sessions will be supervised by each study site independently. This supports a harmonized study design with sufficient flexibility to take cross-cultural differences into account, which appears to be relevant for international living lab research [4]. Each session will adopt the following format: introduction, mapping challenges, demonstration, and reflection and conclusion. The main research topics for cocreation include acceptability, feasibility, usability, facilitators and barriers, advantages and limitations, and implementation, from the perspective of both end users (and informal caregivers) and professionals. These topics will be studied via questions and exercises. The duration of a session is approximately 1.5 to 2 hours. Following cocreation, an intermediate phase (between phases 1 and 2) allows the implementation of minor adjustments to the intervention (eg, duration or frequency of the intervention and content of video vignettes) or protocol (eg, patient-reported outcome measures [PROMs] and patient-reported experience measures [PREMs]) depending on the input that was obtained from professionals, patients, or informal carers during cocreation and discussions among living labs. Note, however, that the intermediate phase might differ among study partners depending on the results of the separate cocreation sessions.

#### Small-Scale Pilot Studies (Phase 2)

##### General Overview

A mixed methods study design will be adopted in which 6 (technological) interventions will be implemented in rehabilitation practice across four countries (Greece, Spain, Belgium, and Canada) to assess user experience and explore the (preliminary) effects of the implemented intervention. Note, however, that not all interventions are technological in nature but that technology will also be used for data collection purposes. The research protocol comprises a variety of study designs, including pre-post designs, controlled studies, or a collaborative participatory research design. However, similar patient samples and data collection instruments and technologies will be adopted across small-scale pilot studies (Textbox 1). For example, general PROMs and PREMs concerning health and well-being include measures of quality of life (eg, 36-Item Short-Form Health Survey [30], World Health Organization Quality of Life Brief Version [31], Patient-Reported Outcomes Measurement Information System–Short Form [version 1.1]) and well-being (eg, 36-Item Short-Form Health Survey [30] and Patient-Reported Outcomes Measurement Information System–Short Form [version 1.1]). In addition, common living lab outcomes include measures of user experience (eg, Weiner scales [32] and System Usability Scale [33]). Note, however, that these PROMs and PREMs might undergo minor adjustments as a consequence of the cocreation process in phase 1 of the study. In addition to these PROMs, study-specific outcome measures are presented in Table 2. The pilot studies not only implement traditional living lab methodology, that is, experience-based (self-report) measures focusing on usability and user experience, but also demonstrate living lab infrastructure, such as wearables and other pieces of measurement equipment. This combination of different data collection methods provides a rich data set to evaluate rehabilitation interventions and share expertise regarding data collection and analysis among living labs.

**Overview of data collection methodology: general data collection.**

**Overview of data collection methodology**

- Demographics (eg, gender, age, marital status, employment status, educational level, and stroke information)
- Anthropometry (eg, weight, height, body mass index, and neck and waist circumference)
- Sleep behavior (eg, sleep hours)
- Smoking behavior (eg, pack years)
- Quality of life (eg, World Health Organization Quality of Life–Age [34], 36-Item Short-Form Health Survey [30], Patient-Reported Outcomes Measurement Information System [35], and the 5-level classification system of the EQ-5D [36])
- Well-being (eg, 36-Item Short-Form Health Survey [30] or Warwick-Edinburgh Mental Well-being Scale [37])
- User experience of patients and clinicians (eg, System Usability Scale [33], User Experience Questionnaire [38], Weiner measures [32], or Usability Metric for User Experience (UMUX) [39])
- Interviews of patients and clinicians

**Table 2:** Overview of data collection methodology: additional pilot study–specific data collection.

Living Lab and intervention and outcomes				Instruments
**AUTH**				
	**Short-arm human centrifuge**			
		Physical activity	Smartwatch (Polar Electro Oy, Kempele, Finland)	
		Cardiovascular activity	ClearSight (Edwards Lifesciences Corporation) noninvasive monitor, CNOGA (CNOGA Tensor tip MTX)	
		Muscle oxygen saturation	Moxy (Fortiori Design LLC)	
		Electroencephalographic data	Neurofax EEG-1200 32-channel device (Nihon Kohden)	
		Neurologic impairment	Expanded Disability Status Scale [40]	
		Mobility	6-minute walk test [41], timed up and go test [42], five times sit-to-stand test [43]	
		Balance	Berg Balance Scale [44], KFORCE (KINVENT), posturography, Dynamic Gait Index [45], backwards walking	
		Cognitive assessment	Symbol Digit Modalities Test [46]	
		Dual tasking	Walking while talking [47]	
**GAIA-Ocean Living Lab**				
	**Art therapy**			
		Physical activity	International Physical Activity Questionnaire for elderly [48] or Global Physical Activity Questionnaire [49]	
		Balance	Berg Balance Scale [44], Activities-Specific Balance Confidence Scale [50], Smart Balance Board (Smartifier Oy)	
		Physiology	Smart devices (Xiaomi Mi Band 5, Samsung Galaxy Watch 3, and CAPTAIN eCoach [51])	
		Stress	Perceived Stress Scale [52]	
**INTRAS**				
	**Gradior Cognitive**			
		Neuropsychological or cognitive assessment	Screening: Montreal Cognitive Assessment [53], Gradior Cognitive	
		Performance characteristics	Gradior Cognitive: % successes, graded cognitive performance scores, mistakes and successes by commission, mistakes by omission, number of performed sessions	
**LiCalab-Mobilab & Care**				
	**Virtual reality mirror therapy**			
		Arm functionality	Fugl-Meyer assessment [54]	
		Pain	Visual analogue scale	
		Range of motion	Wearable wireless T-Sens Motion sensors of the Captiv L7000 (TEA Ergo)	
**UQAM-CRIR**				
	**Teledance**			
		Participation and performance characteristics	Frequency and time of use and active practice time or motor engagement (via OpenTera platform or video recordings)	
		User experience	Short punctual or spot-check surveys (audio and visual or via OpenTera platform)	
**McGILL-UdeM-CRIR**				
	**WALKAGAIN (augmented gait training)**			
		Walking in the community (eg, shopping and visiting a museum)	Qualitative (open-ended question)	
		Single and dual tasking	Assessment of walking (single), walking and reading, and walking in the presence of distractors	
		Movement characteristics (eg, tone, loss of sensation, sensorimotor deficits, and muscle strength)	Inertial measurement units or actigraph units	
		Mobility (eg, balance, gait impairments, and endurance)	Berg Balance Scale [44], 6-minute walk test [41], Mini-Balance Evaluation Systems Test [55], Activities-Specific Balance Confidence Scale [50]	
		Pain	Visual analogue scale	
		Participation	Community Healthy Activities Model Program for Seniors [56]	

##### Short-Arm Human Centrifuge

**Figure 2:**
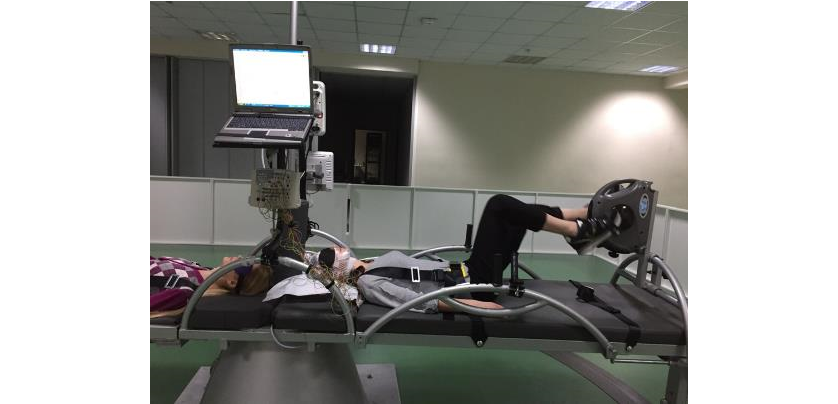
Short-arm human centrifuge.

This small-scale pilot study will be conducted by AUTH in Greece. In this pilot study, the SAHC is used for physical and cognitive rehabilitation to mitigate the detrimental effects of bed rest [20,57] (Figure 2). The objective is to assess the combined effects of artificial gravity and physical activity compared with standard of care in patients with stroke (acute or chronic) and healthy older adults over a period of 3 months (1 hour per session, 3 sessions per week). Participants will be assigned randomly to the SAHC training, standard of care training, or passive control. Data will be collected across the domains of body structure and function, activity, and participation as classified by the World Health Organization International Classification of Functioning, disability, and health, at six time points: at baseline, 4 weeks, 8 weeks, 3 months, and 6- and 12-month follow-ups.

Participants will include 30 patients with acute stroke (15 SAHC and 15 passive control) and 30 patients with chronic stroke (15 SAHC and 15 passive control). They will be recruited from the Euromedica-Arogi Rehabilitation Center in Thessaloniki, Greece. The inclusion criteria include the following: individuals aged 18 to 70 years, without psychiatric disorder, without vertigo, without nausea or chronic pain, and without a history of syncope. The exclusion criteria include the following: individuals with a height >2 m, elite athletes, individuals with chronic use of substances (drugs or alcohol), individuals who have had a recent surgery, individuals with current arrhythmias, individuals with severe migraines, pregnant individuals, individuals with epilepsy, individuals with cholelithiasis or kidney stones, individuals with recent wounds from surgery, individuals with recent fractures, individuals with acute inflammation or pain, individuals with newly inserted metal pins or plates, and individuals with newly implanted stents.

The centrifugation on the SAHC will be combined with mild-intensity exercise based on the maximum heart rate. The SAHC intervention consists of 3 sessions per week, each with a duration of 1 hour, for 3 months. The participant is positioned in a supine and horizontal position on the rotation bed, with the head located toward the center. The beds with the patients turn around the axis of rotation with a force that is the product of body mass, distance from the axis of rotation, and angular velocity squared. Initially, there will be 1 session to familiarize participants with the SAHC group and to individually assess the optimal *g* load according to the participant’s cardiovascular functioning with cardiac output, stroke volume, mean arterial pressure, diastolic blood pressure, systolic blood pressure, and heart rate. These criteria are monitored at each training session and are used to dynamically adapt the intervention intensity. More specifically, after 6 training sessions (2 weeks), the centrifugation load will be increased, and centrifugation will be combined with either aerobic exercise (through an ergometer) or resistance training through elastic training bands during centrifugation (depending on cardiovascular criteria). Further verification of the dynamic configuration of the intervention will be provided by the electroencephalographic assessment. Functional connectivity and cortical-network features derived from graph theory will be used by deep learning algorithms (convolutional neural networks) to define the optimal centrifuge training.

##### Art Therapy

This small-scale pilot study will be conducted by GAIA-Ocean Living Lab in Spain. The objective of this small-scale pilot study is to assess the performing art methodologies (music and dance therapy) in different green and public spaces in the area of Gernika-Lumo (Spain) by assessing their feasibility and to explore their potential impact for individuals with stress-related conditions to support the therapists as well as formal and informal caregivers. Data will be collected at two time points: at baseline and after the end of the intervention.

A sample of 20 healthy men and women will be recruited from Gernika-Lumo (Biscay, Spain). Participants will be included if they are aged 18 to 85 years and will be allocated randomly to the testing or control group. Individuals with a medical history of severe cognitive impairment are not eligible to participate.

The performing arts intervention is a form of physical and mental rehabilitation offered to volunteers in public spaces. Each intervention will be conducted by experts on the methodologies for 2 months, twice a week. Participants will be instructed to follow the different activities, and their biometrics will be monitored during the sessions (smartwatch, smart band, and balance board) to assess their performance and compare their progress. At the end of the pilot period, participants in the control group will also have the opportunity to try out the intervention.

##### Gradior Cognitive

**Figure 3:**
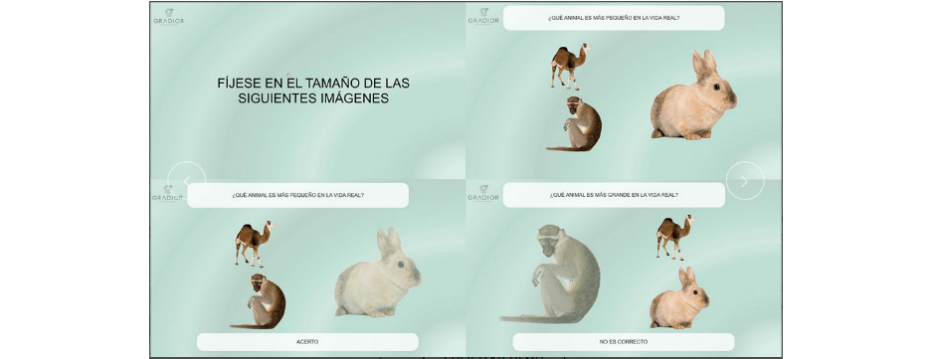
Gradior Cognitive screenshots.

This small-scale pilot study will be conducted by INTRAS in Valladolid, Spain. The objective of this small-scale pilot study is to assess the most recent functionalities of Gradior Cognitive (Figure 3) by exploring the usability and preliminary effectiveness of the new features oriented to support the therapists and by assessing user experience and satisfaction in older adults with MCI. Data will be collected at two time points: at baseline and immediately after the end of the intervention.

Outcome measures and general feedback will be collected from therapists ([clinical] psychologists, neuropsychologists, and gerontologists) using the new version of Gradior Cognitive (N=6 to 10). Furthermore, approximately 30 older adults with MCI will be recruited from the INTRAS Memory Clinic and Neuropsychological Rehabilitation Center in Valladolid, Spain. Potential older participants will be screened (cognitive abilities) before study participation. The inclusion criteria include the following: patients aged ≥55 years; patients with willingness and ability to collaborate in the study; and patients with either (1) a Mini-Mental State Examination [58] score of ≥21 and ≤27 indicating MCI according to the Petersen criteria for MCI-amnestic [59] and the international working group on MCI-amnestic or (2) initial dementia as categorized by the Diagnostic and Statistical Manual of Mental Disorders, fifth edition, and a Clinical Dementia Rating of ≥1 and ≤2 and a Geriatric Depression Scale score of <5 [60]. The exclusion criteria include the following: hearing or visual impairments (inability to use the devices); psychiatric, neurological, or nutritional condition preventing the individual from participating in the study; having a history of substance abuse (alcoholism or alcoholic-type dementia); and being on antipsychotic medication.

Gradior Cognitive is a neuropsychological evaluation and rehabilitation software for the implementation of higher cognitive function training programs for people with cognitive deficit or impairment. Gradior Cognitive allows working on attention, perception, orientation, memory, calculation, executive function, language, and reasoning in adults. The system consists of a website (intended for professionals) and an app for users to conduct intervention sessions. It analyzes and updates the results obtained reporting on users’ performance in different cognitive areas, proposing changes in the session plan. This software supports therapists in the early detection and monitoring of cognitive impairment, also enabling a personalized cognitive rehabilitation program for improvement or maintenance of cognitive skills. Participant therapists included in the study will be using the new Gradior Cognitive features on a daily basis for at least two months. Individual interviews will be conducted to gather details on the therapist’s experience and assess usability and perceived effectiveness. For the older adult participants, the adopted intervention consists of 2-weekly sessions of 30 minutes using Gradior Cognitive for a period of 4 to 6 months. Sessions will be supervised by experienced Gradior professionals.

##### VR Mirror Therapy

This small-scale pilot study will be conducted by LiCalab-Mobilab & Care in Belgium. Mirror therapy is a rehabilitation method during which a mirror is placed between the arms or legs of a patient to create the illusion that a patient’s affected limb is moving when the patient sees his or her unaffected limb moving in the mirror. The objective is to assess the noninferiority of VR mirror therapy as compared with standard mirror therapy in patients with subacute and chronic stroke, as well as to assess user experience of the VR mirror therapy from the perspective of both patients and clinicians. To do so, a two-arm, unblinded, randomized, controlled, parallel design is adopted to compare VR mirror therapy to regular mirror therapy (treatment as usual) in patients with stroke. A total of 20 patients with stroke will be randomly assigned to either the VR group or the control group receiving mirror therapy as usual. Data will be collected at four time points: at baseline, immediately after the first intervention session, before the last intervention session, and immediately after the last intervention session.

A total of 20 patients with stroke will be recruited by clinicians in partner rehabilitation centers or rehabilitation departments of general hospitals in Belgium and will be randomized to VR mirror therapy or treatment as usual. The clinicians themselves will also participate in the study by contributing data on usability and feasibility. The inclusion criteria for patients include the following: patients with stroke in the subacute or chronic phase, aged 18 to 85 years, with normally functioning upper limb on the nonaffected side and impaired functioning of the upper limb on the affected side, with the ability to sit independently on a chair or in a wheelchair in order to freely move the (unaffected) upper limb, and with the ability to follow verbal instructions. The exclusion criteria for patients include the following: patients with acute stroke, medically unstable patients, patients with visual deficits interfering with the execution of activities of daily living, patients with allergies for materials used on the VR headset (eg, silicone), patients with epilepsy, patients who have extreme sensitivity to motion sickness, and patients with facial wounds or lesions impairing the use of the VR headset.

**Figure 4:**
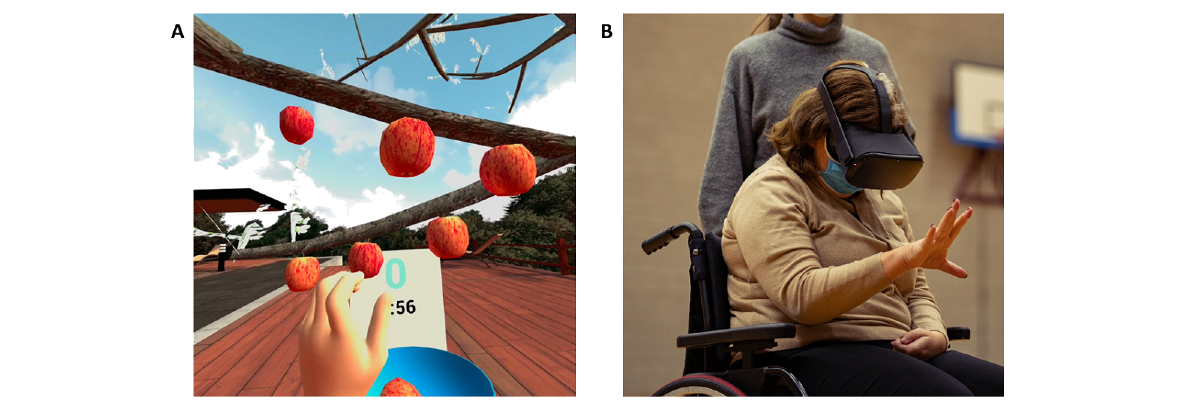
Virtual reality (VR) mirror therapy: (A) screenshot of the apple-picking task from the perspective of a participant and (B) a patient performing the VR mirror therapy.

Using an Oculus Quest VR headset (Meta) with built-in hand tracking, participants will be immersed in a VR kitchen and garden, where they will perform various actions in order to successfully complete a cooking program (Figure 4). These actions must be performed (in reality) by the side of the body that is unaffected by the stroke and include only upper-limb activities of daily living such as grasping ingredients or kitchen utensils, cutting vegetables, putting ingredients in a cooking pot, and stirring in the cooking pot. The movements of the unaffected side are projected to the affected side (mirrored), so that the patient appears to be performing the actions with the affected side. Participants in the control group will perform standard mirror therapy. Both groups will be administered the assigned therapy once per week over a period of 8 weeks.

##### Teledance

This small-scale pilot study will be conducted by UQAM-CRIR in Canada. The objective is to test the teledance intervention, including a series of video vignettes adapted for stroke rehabilitation and follow-up modalities with users (asynchronous and synchronous). To do so, the intervention will be subject to a formative evaluation by the users to identify and resolve problems that may influence their experience [61] and to explore its potential impacts. By testing the intervention within an intensive functional rehabilitation setting, the objective is to identify the characteristics of users to whom such an intervention can be applied, evaluate its usability and safety, and explore its potential impacts. Data will be collected during each intervention session and at the end of the study.

Participants will include 9 clinicians (eg, physiotherapists, occupational therapists, and speech language therapists), 15 patients with stroke, and their relatives (N=15, comprising spouses, adult family members, or caregivers) recruited from 3 rehabilitation sites in Montreal, Canada. Included clinicians are required to have at least one year of work experience in a stroke rehabilitation program. The inclusion criteria for patients include the following: new admission to the facility’s stroke program (≤14 days) and ability to participate safely and independently following an initial assisted teledance session with a clinician (or with the sustained assistance of a relative). Patients who are aggressive or have known symptoms of dementia and Alzheimer disease before the stroke and who do not have sufficient cognitive abilities to follow instructions and give informed consent will be excluded. Relatives will include spouses, adult family members, or caregivers who are in close contact with the patient during rehabilitation. All participants must be able to understand and express themselves in French.

The teledance intervention will be offered to patients during their stay in the hospital and will consist of video vignettes delivered via the OpenTera software platform [62] on a digital tablet (eg, iPad), as well as follow-up modalities offered via OpenTera or other means. The intervention will be conducted by 2 dance educators for 5 to 6 weeks. Video vignettes will be +20 to −20 minutes long and offer thematic content, which will be performed in a sitting or standing position and either alone or assisted. Patients will be instructed to perform teledance at least 6 times per week for approximately 2 hours of dance per week. Follow-up sessions (patient-clinician, patient-dance educator, or clinician-dance educator) will also be included.

##### Augmented Gait Training

This small-scale pilot study (WALKAGAIN) will be conducted by McGILL-UdeM-CRIR in Canada. The main objective of this pilot study is to assess the effect of adding augmented gait training to usual rehabilitation and technology targeting the walking abilities of individuals admitted to rehabilitation after stroke in single-tasking, dual-tasking, and multitasking conditions. A secondary objective is to establish an international common data set for standing mobility assessment and conceptualize the common elements (dose, progression, etc) of the training protocols to be able to compare data among sites or analyze them together. To do so, a before-after pragmatic design with a blind outcome assessment will be set up to assess the performance of participants using augmented training protocols as compared with a control group or previously collected data (if available). The data from two Montreal Metropolitan (Canada) rehabilitation sites (Jewish Rehabilitation Hospital and Institut de réadaptation Gingras-Lindsay-de-Montréal) will be pooled together to assess the overall effect of the concepts of augmented gait training. Standardized outcomes will be collected in the week before and week after the locomotor training. We will also implement a follow-up assessment at 6 months (telephone call).

A total of 12 to 15 patients with stroke will be recruited from the stroke unit of the 2 sites for each locomotor training protocol. Participants will be selected for their potential to improve gait capacities and according to the requirements of their individual goals for improvement. The inclusion criteria will be the following: (1) having a first unilateral stroke and being in rehabilitation, (2) having initiated the gait training with or without assistive devices, (3) tolerating 1 hour of exercise with breaks, (4) in the presence of aphasia showing no more than mild or mild to moderate impairment, (5) having a reliable yes and no. The exclusion criteria will include cerebellar lesions, major pain, hemineglect or hemianopsia, signs of major depression quantified with a score of ≥10 out of 15 on the Geriatric Depression Scale [63], severe cognitive deficits defined with a score of <25 out of 30 on the Mini-Mental State Examination developed by Folstein et al [64], cardiorespiratory problems, and other medical and cognitive conditions that could affect the ability to understand instructions, as verified by medical records. We will also collect data on usability, feasibility, and opinions from clinicians about the training interventions through questionnaires or interviews.

**Figure 5:**
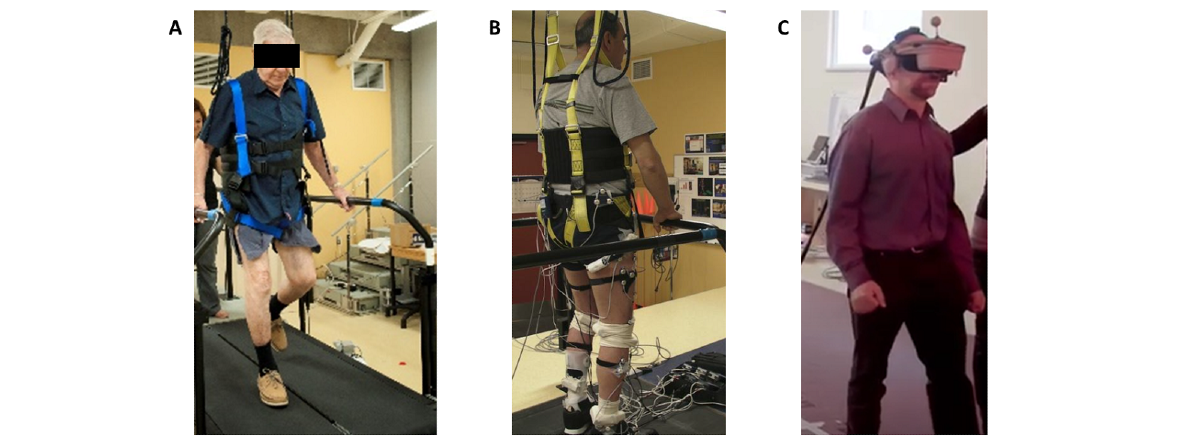
Examples of technologies for augmented gait training: (A) split-belt training (biomechanics), (B) vibrations (haptic), and (C) virtual reality.

Three major technologies (either individually or combined) will be used for locomotor training: biomechanical (eg, split belt training, walking with loaded segment, ascending slope), haptic (eg, vibrations), and VR (virtual environment), as shown in Figure 5. The precise locomotor training protocol will be decided by the clinicians in consultation with the researchers and will take into consideration the preferences of the participants (after phase 1). All locomotor training will include cognitive or language tasks as well attention-demanding tasks, implemented gradually from simple to more complex (single tasking, dual tasking, and multitasking) while the gait training will also be progressed. The training will consist of, for example, 30 minutes of walking or doing a number of steps, using the selected protocol with 5 minutes of warming-up and 5 minutes of recovery. Participants will be allowed to take breaks if their heart rate exceeds 60% of their maximal heart rate or they reported an exertion of 5 on the Borg rating scale, which represents severe exertion [65]. The training will be restarted when the participant’s heart rate will lower back to its level at rest. The augmented gait training will be given twice a week for 10 sessions (5 weeks). Before each training session, the participant’s heart rate (eg, Polar Electro Oy) and blood pressure will be taken at rest. The age-related maximum heart rate will be calculated using the Karvonen formula [66].

#### Harmonization (Phase 3)

To define harmonized protocols and procedures (eg, for patient screening=outcomes and outcome measures) for living lab research in the context of technology in rehabilitation, the consensus process will involve a modified Delphi methodology (web-based, 3-round Delphi survey) followed by a consensus meeting of the living labs. The Delphi technique is a method for reaching consensus comprised of sequential questionnaires answered anonymously by a panel of experts with feedback provided after each questionnaire round [67,68]. A preliminary list of items for the Delphi survey will be based on literature research and findings of phases 1 and 2. For each living lab, 2 researchers participated (N=12).

### Data Analysis

#### Cocreation (Phase 1)

Best practices for content analysis will be exchanged among partners to ensure a harmonized approach and the generalizability of the findings. Subsequently, for each test site, one or more researchers trained in qualitative methodology will provide a written report of each cocreation workshop and will use content analysis to analyze the data using main themes and subthemes.

#### Small-Scale Pilot Studies (Phase 2)

Best practices for analyses will be exchanged among partners to ensure a harmonized approach and the generalizability of the findings. However, for each small-scale pilot study, the statistical approach might vary according to the needs of the respective study. In general, descriptive statistics will be presented for demographic information. For quantitative data, normal distribution will be tested. The effects of an intervention on dependent variables will be assessed over time (eg, before-after, follow-up sessions) using repeated-measures parametric or nonparametric tests. The effect sizes of changes found among evaluations will be computed. The significance level is set at *P*<.05.

#### Harmonization (Phase 3)

Via the Delphi method, the living labs will reach consensus on which procedures (ie, outcomes, outcome measures, and devices) to use in a rehabilitation context. Each outcome, outcome measure, and device will be represented as an item in a list. In Delphi survey round 1, the international panel of living lab experts will be instructed to rate the importance of each item and asked to provide new items or suggest revisions of existing items. Consensus will be reached when at least 70% of the panel strongly agree or disagree on inclusion or exclusion of an item. Items that did not reach agreement and newly suggested items will be taken to round 2, together with feedback of round 1 responses. After the second round, items will be ranked by importance, and a list of items will be created. This list will be distributed among the panel members to discuss during a final consensus meeting.

### Ethical and Legal Considerations

On a lower level, each partner institution involved in this study will submit an institutional review board application or ethical committee application according to the respective national regulations at the latest in February 2022. Informed consent will be obtained from all participants before data collection. For the pilot study involving individuals with (mild) cognitive disabilities, a team of trained psychologists will conduct recruitment of participants to ensure that potential participants’ cognitive functions and comprehension abilities allow them to provide written informed consent. Only pseudonymized data will be shared among the living labs in the context of harmonization of procedures and protocols.

On a higher level, with the VITALISE project, we also aim to collect information on differences and similarities concerning legal frameworks, data management, and privacy. To date, the following project deliverables have addressed the issues of legal frameworks, privacy, and data management: Deliverable *D1.2 First Version of Ethics and Safety Manual* (submitted to European Commission on September 29, 2021) reports on the VITALISE project’s international, European, and national ethical regulations, device standards, and certifications as well as accepted data management procedures; presents a first plan for the VITALISE scheduled data collection to be compliant with the reported regulations and guidelines; and evaluates potential concerns. In addition, project deliverable *D1.4 Data Management Plan (first version)* (submitted to European Commission on September 29, 2021) provides provisory information regarding the data to be collected during the project, addresses the issue of data reuse for further exploitation by expanding on the manner in which the data will be made accessible, and gives information on the data’s curation and preservation. Deliverables will be available to the general public via the VITALISE project website [1] after completion of the European Commission review process (approximately October 2022). Concerning informed consent procedures, identification and recruitment of participants, security measures, and anonymization or pseudonymization procedures of personal data and data transfer to non-EU countries, two project deliverables *D14.1 H—Requirement No. 1* and *14.2 Protection of Personal Data—Requirement No. 3* have been submitted to the European Commission on November 29, 2021. These deliverables will, however, only be available to the consortium members and Commission services.

## Results

Research activities (eg, participant enrollment, data collection, and data analysis) for this project will start in March 2022 (phase 1) and June 2022 (phase 2) and end in March 2024. Phase 1 (cocreation) will provide insights into different stakeholders’ views (ie, end users, clinicians, and informal caregivers) on the role of technology in rehabilitation on a European level (and beyond), as well as specific suggestions regarding rehabilitation innovations. Phase 2 (small-scale pilot studies) will provide insights into end user experiences, and usability and preliminary effectiveness of 6 selected neuropsychological or physical rehabilitation interventions for patients with stroke and older individuals. Phase 3 (harmonization via the Delphi methodology) will provide a consensus on harmonized screening, outcomes, and outcome measures for living lab research in the context of technology in rehabilitation. Together, these research activities will provide an opportunity for all involved living labs (and beyond) to gain knowledge on specific technologies intended for rehabilitation and to discuss and adopt each other’s technologies or procedures, to harmonize living lab procedures and infrastructures concerning the use of innovative technology in rehabilitation on a European level.

## Discussion

### Study Significance

This JRA protocol needs to be considered in light of the European Commission’s Horizon 2020 program called *Integrating Activities for Starting Communities* (INFRAIA-02-2020). This JRA is part of three JRAs included in the VITALISE project that will investigate three domains of health and well-being research, namely, rehabilitation, transitional care, and everyday living environments. With these JRAs, the VITALISE project aims to improve, in quality or quantity, the integrated services provided at a European level by serving as test beds for harmonizing procedures and services. This JRA, in particular, focuses on the use of technology in rehabilitation.

The primary outcome of this study (JRA) allows the involved living labs to exploit similarities in existing services and infrastructures, such as devices, hardware, and software being used for either intervention or data collection purposes, and harmonize (and potentially eliminate) identified differences. The secondary outcome of this JRA is an increased knowledge and improvement of technological innovations for rehabilitation of patients with stroke and individuals living with MCI or dementia, on an international level.

The study design involves a collaboration of multiple international living labs (1) to gain insight into stakeholders’ views on the use of innovative technologies for rehabilitation on an international level, (2) to explore the (preliminary) effects of the pilot intervention, and (3) to explore and learn from each living lab by using each other’s infrastructures, to harmonize procedures and infrastructures across living labs.

### Conclusions

The JRA described in this research protocol demonstrates cocreation activities and implements a variety of study designs that illustrate living lab practices and infrastructure. The knowledge gained by the execution of this research will lead to harmonized procedures and protocols in a rehabilitation context for living labs in Europe and beyond. In addition, we will also be able to provide new insights for improving the implementation of innovative technologies in rehabilitation.

## Abbreviations

JRA: joint research activity
MCI: mild cognitive impairment
PREM: patient-reported experience measure
PROM: patient-reported outcome measure
SAHC: short-arm human centrifuge
VITALISE: Virtual Health and Wellbeing Living Lab Infrastructure
VR: virtual reality
